# Gamma probe and ultrasound-guided fine needle aspiration cytology of the sentinel node (GULF) trial

**DOI:** 10.1007/s00259-018-4014-3

**Published:** 2018-04-26

**Authors:** Daniëlle Verver, Charlotte M. C. Oude Ophuis, Lisa B. Koppert, Cécile de Monyé, Carolien H. M. van Deurzen, Senada Koljenović, Annemarie Bruining, Bernies van der Hiel, Sylvia ter Meulen, Alexander C. J. van Akkooi, Cornelis Verhoef, Dirk J. Grünhagen

**Affiliations:** 1 Department of Surgical Oncology, Erasmus MC Cancer Institute, Groene Hilledijk 301, 3075 EA Rotterdam, The Netherlands; 2 Department of Radiology, Erasmus MC Cancer Institute, Groene Hilledijk 301, 3075EA Rotterdam, The Netherlands; 3 Department of Pathology, Erasmus Medical Center, Wytemaweg 80, 3015 CN Rotterdam, The Netherlands; 4 Department of Radiology, Netherlands Cancer Institute – Antoni van Leeuwenhoek, Plesmanlaan 121, 1066 CX Amsterdam, The Netherlands; 5 Department of Nuclear Medicine, Netherlands Cancer Institute – Antoni van Leeuwenhoek, Plesmanlaan 121, 1066 CX Amsterdam, The Netherlands; 6 Department of Surgical Oncology, Netherlands Cancer Institute – Antoni van Leeuwenhoek, Plesmanlaan 121, 1066 CX Amsterdam, the Netherlands

**Keywords:** Sentinel lymph node biopsy, Gamma probe, Ultrasound, Fine needle aspiration cytology, Melanoma, Breast cancer

## Abstract

**Purpose:**

Sentinel lymph node biopsy (SLNB) was introduced as a minimally invasive technique for nodal staging. Since associated morbidity is not negligible, it is highly relevant to pursue a more minimally invasive alternative. The purpose of this study was to prospectively evaluate the sensitivity of fine needle aspiration cytology (FNAC) with combined gamma probe and ultrasound (US) guidance in comparison with the gold standard histology of the sentinel node (SN) after SLNB for detecting metastasis.

**Methods:**

The study was designed as a prospective, multicentre, open-label, single-arm trial enrolling patients with newly diagnosed cutaneous melanoma or breast cancer between May 2015 and August 2017. Sample radioactivity was measured using a Mini 900 scintillation monitor. After FNAC, all patients underwent SLNB. Sensitivity, specificity, positive predictive value (PPV) and negative predictive value (NPV) were estimated.

**Results:**

Accrual was terminated early following an unplanned interim analysis indicating that a FNAC sensitivity of at least 80% could not be achieved. In total 58 patients of the originally planned 116 patients underwent FNAC with gamma probe and US guidance. There were no true-positive FNAC results, 14 false-negative results and one false-positive result, and thus the sensitivity, specificity, PPV and NPV of FNAC were 0%, 98%, 0% and 75%, respectively. At least 75% of the FNAC samples had a radioactivity signal higher than the background signal.

**Conclusion:**

FNAC with gamma probe and US guidance is not able to correctly detect metastases in the SN and is therefore not able to replace SLNB. Gamma probe-guided US is a highly accurate method for correctly identifying the SN, which offers possibilities for future research.

**Electronic supplementary material:**

The online version of this article (10.1007/s00259-018-4014-3) contains supplementary material, which is available to authorized users.

## Introduction

The sentinel lymph node biopsy (SLNB) procedure was introduced in the early 1990s as a less-invasive technique than elective lymph node dissection, enabling selective detection and histopathological inspection of the primary draining lymph node in the regional lymph node basin related to the primary tumour site, e.g. melanoma or breast cancer [1, 2]. The status of the (sentinel) lymph nodes is one of the most important prognostic indicators for recurrence and survival [3–5]. In addition, (sentinel) lymph node status guides locoregional treatment decisions and will probably soon guide the choice of systemic treatment.

Although less invasive than elective lymph node dissection, the morbidity associated with SLNB is not negligible. This is of particular concern since a positive sentinel node (SN) is found in only 20–30% of patients [5–7]. Morbidity occurs in approximately 11% of patients, with the most common early postoperative complications being seroma (about 5%) and infection (about 3%) [8]. Lymphoedema has been reported to occur in at least 6% of patients [9, 10]. It is important to note that SLNB does not improve survival but only provides accurate and important staging information [5, 11]. In this light, it seems highly relevant to pursue a more minimally invasive alternative to SLNB. Fine needle aspiration cytology (FNAC) with ultrasound (US) guidance may provide a good minimally invasive alternative. Several studies have focused on US examination with or without FNAC in melanoma patients, but sensitivity rates vary greatly and most studies lacked a method to accurately identify the SN prior to US examination and FNAC [12]. This problem could be overcome by using a hand-held gamma probe as an aid to US identification of the SN after lymphoscintigraphy. This has been shown to be feasible in several studies in breast cancer patients, in which the SN was correctly identified in 75–100% of patients [13–16].

The purpose of this study was to prospectively evaluate the sensitivity of FNAC with combined gamma probe and US guidance compared with the gold standard, histology of the SN after SLNB, for detecting SN metastasis.

## Materials and methods

### Study design

Details of the study design and protocol have been published previously [12]. Briefly, the trial was designed as a prospective, multicentre, open-label, single-arm trial and was performed in two Dutch hospitals. The Ethical Review Board approved the study protocol. This trial was registered with The Netherlands Trial Registry (NTR; ID NRT5193, 1 May 2015). The study was prepared in accordance with the Standards for Reporting of Diagnostic Accuracy Studies [17].

### Participants

Patients with newly diagnosed cT1b-4N0M0 cutaneous melanoma or cT1-3N0M0 breast cancer were recruited from May 2015 to August 2017. Patients were excluded if they were unwilling or unable to give informed consent, or if they had a clinically suspicious lymph node, other known malignancy with potential to disseminate to the axillary or groin lymph node basins, prior lymph node biopsy, or no SN visible on lymphoscintigraphy. All participants provided written informed consent.

### Procedures

**Fig. 1:**
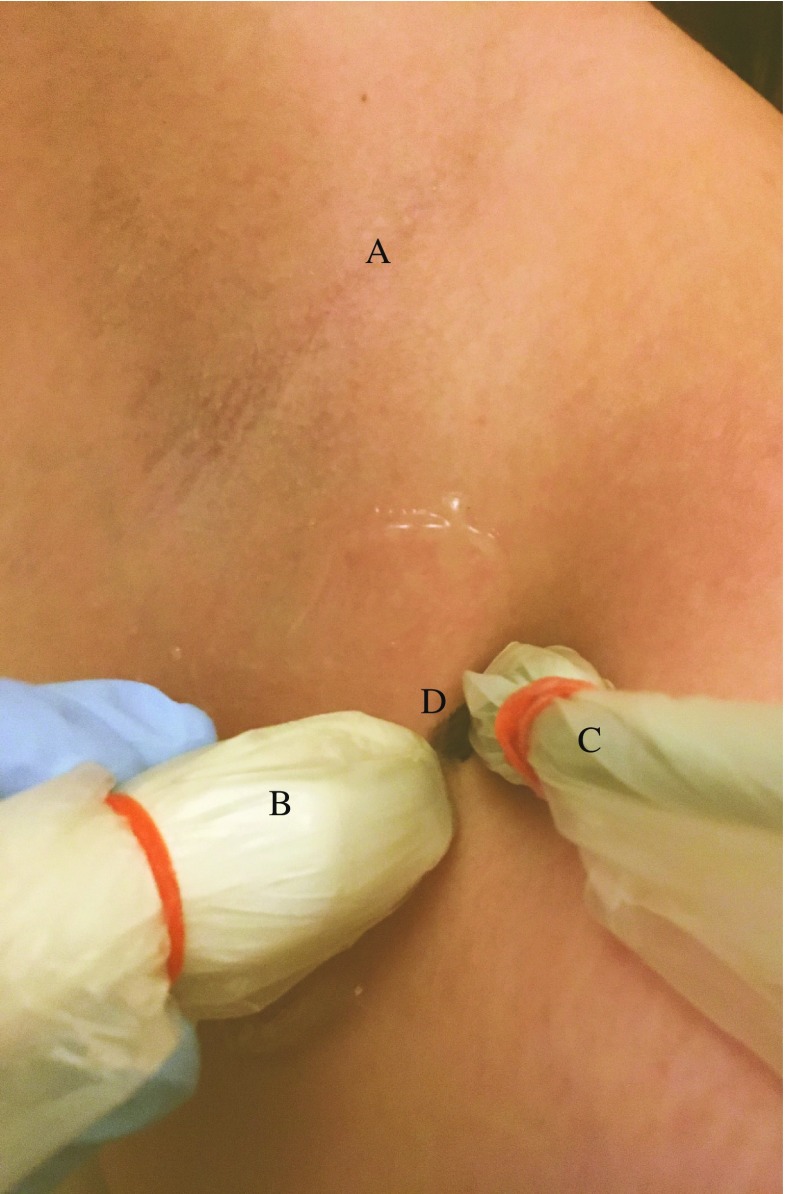
Identification of the presumed sentinel node in the axilla (*A*) using the ultrasound probe (*B*), gamma probe (*C*) and the skin mark (*D*)

After peritumoral or intradermal injection of ^99m^Tc-nanocolloid, lymphoscintigraphy was performed according to the institution’s standard protocol during the 24 h before surgery to define the location of the SN. A nuclear medicine specialist reported information regarding the identified SN basin(s) and primary tier SN(s). The presumed SN(s) were distinguished from the second-tier nodes by visualization of the first node or a direct drainage pathway. Following successful lymphoscintigraphy, a dedicated radiologist identified the hot spot over the skin using a hand-held gamma probe (16 mm Europrobe 3) and the area was examined using US (Aloka ProSound alpha10) with a 1–15 MHz linear transducer to attempt to visualize the assumed SN (a visible lymph node at the centre of the hotspot as identified with the gamma probe; Fig. 1). Fine needle aspiration of all visualized assumed SN(s) was performed using a 21-gauge needle, regardless of suspicion of metastasis on US examination, with usually one or two cortical samples per SN (depending on the visual yield of each sample). A Mini 900 scintillation monitor with a sodium iodide crystal was used, when available, to measure radioactivity of the samples. All FNAC samples were subsequently transported to and analysed in the pathology laboratory of the Erasmus MC Cancer Institute.

Cytological smears were prepared according to a standard protocol. Cytomorphology was assessed on haematoxylin and eosin (H&E) stained smears. The remainder of the aspirate was expressed into a CytoLyt solution from which a Cellient cell block was prepared, provided that an adequate amount of material was obtained. Cytomorphology was assessed again. In addition immunohistochemical staining was performed using S-100 and Melan-A for melanoma samples and Ker8-18 for breast cancer samples. US examination findings are reported according to the Berlin morphological criteria [18]. The SN identified on US examination was regarded as malignant when the lymph node appeared “balloon shaped”, and suspicious if peripheral perfusion, loss of central echoes, asymmetrical broadening of the parenchyma, or an echo-poor island within an otherwise normal lymph node was present [18, 19]. After FNAC, all patients proceeded directly to the operating room for SLNB. The SNs were handled and assessed in each centre according to the European Organization for Research and Treatment of Cancer (EORTC) SN pathology protocol [20].

### Outcomes

The primary objective of the trial was to assess the sensitivity of FNAC with combined gamma probe and US guidance in detecting SN metastasis in patients with melanoma or breast cancer. Prespecified secondary end-points evaluated in the pilot phase of the study included the SN identification rate and the histological results of core needle biopsy (CNB) in comparison with FNAC and SLNB.

### Statistical analysis

Power and sample size calculations are described in detail elsewhere [12]. Considering a 30% prevalence, the required sample size was 116 patients to detect metastatic SN(s) with a sensitivity of 90% and a 95% confidence interval (CI) of 80–100%, with a two-sided significance level *α* of 0.05 and a power 1 − *β* of 0.80. The estimated recruitment goal was 120 patients.

Negative FNAC was defined as absence of metastatic tumour cells (i.e. negative cytology) or unrepresentative cytology due to collection of an insufficient number of cells. Based on comparison with final histology, four types of FNAC results were defined: false-negative, true-negative, false-positive and true-positive. The same definitions were applied to the CNB results and the US results according to the Berlin criteria. A malignant or suspicious SN on US examination according to the Berlin criteria was recorded as positive.

Continuous data are presented as medians with interquartile ranges (IQR) and categorical data as frequencies with their respective percentages. The number of true-positives, false-positives, false-negatives and true-negatives were calculated. Positive predictive value, negative predictive value, specificity and sensitivity were estimated. When clinical data were missing, the clinical characteristic was categorized as “unknown”. Two-sided *P* values <0.050 were considered statistically significant. SPSS version 22.0 (IBM, Armonk, NY) was used for all statistical analyses.

## Results

**Fig. 2:**
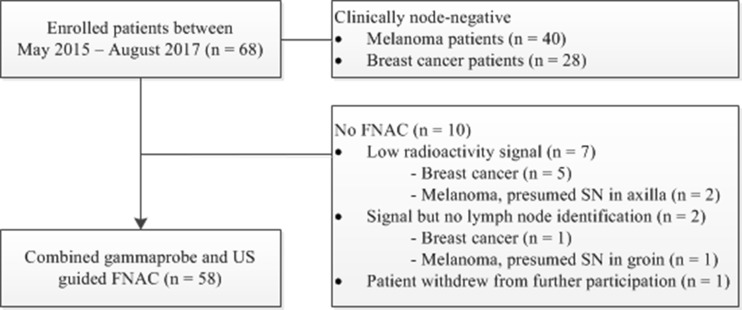
Selection of patients who underwent FNAC with gamma probe and US guidance

A total of 68 clinically node-negative patients with melanoma or breast cancer were enrolled, of whom 10 (15%) did not undergo FNAC and therefore were excluded from further analysis (Fig. 2). Baseline characteristics of the 58 patients who did undergo FNAC with combined gamma probe and US guidance are shown in Table 1. The median number of identified SNs on lymphoscintigraphy was 1 (IQR 1–2) and the median number of SN basins on lymphoscintigraphy was 1 (IQR 1–1). The median number of identified SNs on FNAC with gamma probe and US guidance was 1 (IQR 1–2) and the median number of retrieved SNs during SLNB was 2 (IQR 1–3; *P* < 0.001).

**Table 1:** Baseline characteristics of the 58 analysed patients and tumours

Characteristic	Value
Gender	
Male	18 (31)
Female	40 (69)
Age (years)	56 (44–64)
Body mass index (kg/m^2^)	25.1 (22.4–27.1)
Breast cancer	21 (36)
Size (mm)	15.0 (6.5–28.3)
Side	
Right	14 (67)
Left	7 (33)
Quadrant	
Left upper	8 (38)
Left lower	3 (14)
Medial lower	3 (14)
Medial upper	4 (19)
Histology	
Ductal	15 (71)
Other	6 (29)
Oestrogen receptor status	
Negative	3 (14)
Positive	16 (76)
Unknown	2 (10)
Progesterone receptor status	
Negative	5 (24)
Positive	14 (67)
Unknown	2 (10)
Her2neu status	
Negative	15 (71)
Positive	4 (19)
Unknown	2 (10)
Melanoma	37 (63.8)
Breslow thickness (mm)	1.85 (1.13–4.00)
Location	
Arm	8 (21.6)
Leg	14 (37.8)
Trunk	15 (40.5)
Histology	
Superficial spreading	18 (48.6)
Nodular	10 (27.0)
Acral lentiginous	2 (5.4)
Lentigo maligna	2 (5.4)
Other	2 (5.4)
Unknown	3 (8.1)
Ulceration	
Present	10 (27.0)
Absent	25 (67.6)
Unknown	2 (5.4)
Regression	
Complete	1 (2.7)
Partial	2 (5.4)
Absent	32 (86.5)
Unknown	2 (5.4)
Micro satellites	
Present	1 (2.7)
Absent	33 (89.2)
Unknown	3 (8.1)

The values presented are number (%) or median (IQR)

Originally 116 patients were planned to be included. Slow accrual prompted extension of the predefined study period, but before this was done, an unplanned (non-protocol-specified) interim efficacy analysis was carried out. Evaluation of the results indicated that even in the best case scenario a FNAC sensitivity of at least 80% could not be achieved, and accrual was therefore terminated. Early termination was not related to any safety concerns related to FNAC with gamma probe and US guidance. The interim analysis was performed in July 2017 with available data on the first 53 patients who had undergone FNAC (an additional five patients had been included but data on these patients were not yet complete). The technique was considered successful if a sensitivity of 90% with an upper and lower 95% CI of 80–100% was achieved. Calculations based on the data available showed that in the best-case scenario, the highest achievable sensitivity would be 63% (Supplementary material).

### Results of FNAC with gamma probe and US guidance

The cytological smears were representative in 51 of 58 patients (88%) and additional cytomorphology and immunohistochemistry analysis on Cellient blocks was possible in 39 of 58 patients (67%). In patients with insufficient cytology FNAC was recorded as negative. One of these patients turned out to have a positive SN on histology, and the FNAC result was recorded as false-negative. The radioactivity signal of the material, measured using the Mini 900 scintillation monitor with a sodium iodide crystal, was reported to be more than twice the background signal in 33 of the 44 tested samples (75%).

The results of the FNAC, US examination (according to the Berlin criteria) and CNB and measures of diagnostic accuracy are shown in Table 2. A positive SN on histology after SLNB was found in 14 patients (24.1%), 10 with melanoma and 4 with breast cancer. Submicrometastases were present in 5 of 14 patients (35.7%), 3 with melanoma (<0.1 mm at any site or 0.4 mm subcapsular) and 2 with breast cancer (≤0.2 mm isolated tumour cells). Micrometastases were present in four patients (28.6%), 3 with melanoma (>0.1–1.0 mm) and 1 with breast cancer (>0.2–2.0 mm). Macrometastases were present in the remaining five patients (35.7%), 4 with melanoma (>1.0 mm) and 1 with breast cancer (>2.0 mm).

**Table 2:** Measures of diagnostic accuracy of US examination, FNAC and core needle biopsy

	Results, *n* (%)		
	FNAC (*n* = 58)	US examination (*n* = 58)^a^	CNB (*n* = 10)
True positive	0	2 (3)	0
Tumour			
Breast cancer	n/a	1 (50)	n/a
Melanoma	n/a	1 (50)	n/a
SN location			
Axilla	n/a	1 (50)	n/a
Groin	n/a	1 (50)	n/a
False positive	1 (2)	6 (10)	0
Tumour			
Breast cancer	0	2 (33)	n/a
Melanoma	1 (100)	4 (66)	n/a
SN location			
Axilla	1 (100)	6 (100)	n/a
Groin	0	0	n/a
True negative	43 (74)	38 (66)	9 (90)
Tumour			
Breast cancer	17 (40)	15 (40)	9 (100)
Melanoma	26 (61)	23 (61)	0
SN location			
Axilla	36 (80)^b^	29 (76)	9 (100)
Groin	9 (20)	9 (24)	0
False negative	14 (24)	12 (21)	1 (10)
Tumour			
Breast cancer	4 (29)	3 (25)	1 (100)
Melanoma	10 (71)	9 (75)	0
SN location			
Axilla	6 (43)	5 (42)	1 (100)
Groin	8 (57)	7 (58)	0
Measures, % (95% CI)			
Sensitivity	0	14 (3–38)	0
Specificity	98 (90–100)	86 (74–94)	100
Positive predictive value	0	25 (5 – 59)	0
Negative predictive value	75 (63–85)	76 (63–86)	90 (63–99)

*CI* confidence interval, *CNB* core needle biopsy, *FNAC* fine needle aspiration cytology, *n/a* not applicable, *US* ultrasound

^a^According to the Berlin criteria, a malignant/suspicious sentinel node on US examination was recorded as positive

^b^Two patients had SNs identified in the groin and axilla

In the pilot phase of the study an additional CNB of the SN was done in ten breast cancer patients. The biopsy was not representative in four patients due to collection of an insufficient amount of tissue. Histology after SLNB revealed a macrometastasis in one patient.

## Discussion

The pilot phase of this prospective trial showed that gamma probe-guided US can accurately identify the SN in up to 90% of patients. This is in line with previous reported correct identification rates of 75–100% [13–16]. Furthermore, at least 75% of the FNAC samples had a radioactivity signal more than twice the background signal, which supports the high accuracy of SN identification. However, FNAC lacked sensitivity as it was not able to correctly detect metastases in the SN.

A high body mass index, a high background signal, the presence of a cluster of multiple nodes and a presumed SN location close to the injection site might hamper SN visualization and identification. In the present study, accurate radiographic SN visualization and identification was impossible in 10 of 68 patients (15%). This was predominantly caused by a low transcutaneous radioactivity signal, presumably as a result of poor tracer uptake or tracer migration. SLNB was successful in all ten patients and in at least two patients the signal was also recorded as low during the surgical procedure.

All 51 patients (88%) with representative cytological smears stained with H&E showed normal cytomorphology. Additional cytomorphology and immunohistochemistry analysis on Cellient blocks was possible in 39 patients (67%). One of these patients showed abnormal morphology with positive immunohistochemistry in the Cellient block, and was recorded as having a positive FNAC. However, on histology the SLNB turned out to be negative, even after additional slides had been reviewed. Thus, this FNAC was recorded as false-positive. In 15 patients (39%) the H&E-stained smears and Cellient blocks showed normal morphology but a non-specific immunohistochemistry. The FNAC in these patients was regarded and recorded as negative. Remarkably, histology after SLNB showed metastasis in 6 of these 15 patients (40%).

The Mini 900 scintillation monitor with a sodium iodide crystal was used on 44 samples, and showed a signal more than twice the background signal in 75% of the samples. This supports the high accuracy of SN identification using FNAC with gamma probe and US guidance, as was previously demonstrated in the trial phase with a correct identification rate of 90% [12]. A signal equal to or lower than the background count does not necessarily mean that the sample is not radioactive, but could be a result of a higher background count that occurs occasionally or a low sample volume. So with respect to the false-positive FNAC, the degree of certainty that the lymph node from which FNAC was done was similar to the excised lymph node is at least 75% tending towards 90%. Fusion of US guidance with free-hand SPECT connected to a gamma detection device might become an alternative method that could further increase the correct identification rate of the SN [21].

The FNAC technique would be considered successful when a sensitivity of 90% was achieved. This was based on the assumption that submicrometastases in melanoma (i.e. <0.1 mm at any site or 0.4 mm subcapsular) or isolated tumour cells in breast cancer (i.e. <0.2 mm) are less likely to be detected using FNAC and occur in approximately 10% of patients. Though our hypothesis for detecting positive SNs using FNAC with combined gamma probe and US guidance seemed promising, unfortunately the technique failed, as micrometastatic and also macrometastatic lesions were not detected. This failure can partially be explained by the relatively small sizes of the metastatic lesions in the SN in nine of 14 patients (<1.0 mm for melanoma and <2.0 mm for breast cancer) and in five of these patients the lesions were even submicrometastases. The histological pattern of a subcapsular metastasis is often small and spread in line with the capsule, thus presenting the physician performing the FNAC with a great challenge since it is easy to puncture through the lesion into the parenchyma [22]. On the other hand, a detection limit (the smallest diameter of SN melanoma metastasis that can be detected) for a positive FNAC of 0.3 mm has been reported [23]. This suggests that the detection of micrometastases, and certainly macrometastases, should be possible.

In the previous promising studies on US-guided FNAC almost all procedures were performed by the same senior sonologist who usually obtained four cortical samples per SN [18, 19, 22, 23]. In our study the procedure was performed by several dedicated radiologists with usually one or two cortical samples per SN. Thus, operator dependency and fewer cortical samples per SN (which increases the chance of missing smaller areas of interest within the SN) might also be possible explanations for our results. In this light, the use of large-lumen needles (10-gauge or 12-gauge) might increase the effectiveness of the procedure in detecting metastasis [24]. However, we performed CNB using a 14-gauge needle in ten patients in the pilot phase, and of these patients one turned out to have macrometastasis in the SN which was not detected by CNB. This suggests that piecemeal sampling, regardless of the needle lumen, compromises pathological evaluation. Technical difficulties could also have contributed (e.g. small node, difficult location and/or recognition of the presumed SN, blood in the SN after the first sample). This illustrates the difficulty in the broad implementation of the technique. It is noteworthy that in most previous studies investigating the sensitivity of US-guided FNAC, FNAC was performed only if there was suspicion of metastasis on US examination [12]. This increases the likelihood of a positive FNAC considerably and explains some of the higher reported sensitivity rates, and might explain our sensitivity rate since we performed FNAC regardless of suspicion of metastasis on US examination. Nonetheless, low sensitivity and moderate negative predictive values remain an issue [25].

Our study clearly had some limitations mainly due to the premature termination of the trial. This naturally resulted in a smaller number of included patients than initially planned. Nevertheless, it represents prospectively collected data and the interim analysis showed that even in the best case scenario it would have been impossible to achieve the desired sensitivity of at least 80%. Thus, continuation of the trial would not have led to substantially different conclusions.

Although the main outcome of this trial was negative and was not in accordance with our hypothesis, valuable information was obtained. FNAC with gamma probe and US guidance is not able to correctly detect metastases in the SN and the technique used is therefore not able to replace the SLNB procedure. On the other hand, gamma probe-guided US was found to be highly accurate in correctly identifying the SN. This offers possibilities for evaluating other minimally invasive techniques that incorporate gamma probe-guided US for SN identification.

## Electronic supplementary material

ESM 1 (DOCX 13 kb)
