# Supplementary material for: Gamma probe and ultrasound-guided fine needle aspiration cytology of the sentinel node (GULF) trial
Source: Eur J Nucl Med Mol Imaging. 2018 Apr 26;45(11):1926–33. doi: 10.1007/s00259-018-4014-3 (PMC6132503; doi:10.1007/s00259-018-4014-3)
Supplement: Supplementary file 1 — (DOCX 13 kb) [file 259_2018_4014_MOESM1_ESM.docx]

**Appendix**

*Results Interim Analysis*

The calculated required sample size was 116 patients with a two-sided significance level α = 0.05, and power 1 - β of 0.8, considering a 30% prevalence of metastatic SN(s) to detect a sensitivity of 90% with a 95% confidence interval of 80-100%.

A prevalence of metastatic SNs of 30% in a sample size of 116 patients would result in 35 metastatic SNs. At the time of the unplanned interim analysis (July 2017), 53 patients had undergone gamma probe and US guided FNAC with complete data. All patients were recorded having a negative FNAC. Of these, 13 patients turned out to have a positive SN on histology after SLNB and were thus recorded as having a false negative FNAC. In a best case scenario, you would expect another 22 patients with a positive SN on histology (35 – 13) who would all have a true positive FNAC. In that case, the maximum achievable sensitivity would be 63% (22 / 35). This is lower than the estimated 80% lower bound of the desired 90% sensitivity to regard the technique as being successful.
